# Specific patterns of *gyr*A mutations determine the resistance difference to ciprofloxacin and levofloxacin in *Klebsiella pneumoniae* and *Escherichia coli*

**DOI:** 10.1186/1471-2334-13-8

**Published:** 2013-01-07

**Authors:** Yingmei Fu, Wenli Zhang, Hong Wang, Song Zhao, Yang Chen, Fanfei Meng, Ying Zhang, Hui Xu, Xiaobei Chen, Fengmin Zhang

**Affiliations:** 1 Department of Microbiology, Heilongjiang Province Key Laboratory for Immunity and infection, Pathogenic biology, Bio-pharmaceutical Key Laboratory of Ministry of Education, Harbin Medical University, 157 Baojian Road, Harbin, 150086, China; 2 Department of General Surgery, Minimally Invasive Surgery, First Affiliated Hospital, Harbin Medical University, Harbin, 150010, China; 3 Bio-pharmaceutical Key Laboratory of Ministry of Education, Harbin Medical University, Harbin, China; 4 Current address: The First Hospital of Qiqihaer City, 30 Gongyuan Road, Qiqihaer, 161005, China

**Keywords:** *Enterobacteriaceae*, Fluoroquinolone resistance, *Gyr*A

## Abstract

**Background:**

Wide use of ciprofloxacin and levofloxacin has often led to increased resistance. The resistance rate to these two agents varies in different clinical isolates of *Enterobacteriaceae*. Mutations of GyrA within the quinolone resistance-determining regions have been found to be the main mechanism for quinolone resistance in *Enterobacteriaceae*. It has been shown that only some of the mutations in the *gyr*A gene identified from clinical sources were involved in fluoroquinolone resistance. Whether different patterns of *gyr*A mutation are related to antimicrobial resistance against ciprofloxacin and levofloxacin is unclear.

**Methods:**

The minimum inhibitory concentration (MIC) of ciprofloxacin and levofloxacin were determined by the agar dilution method followed by PCR amplification and sequencing of the quinolone resistance determining region of *gyr*A to identify all the mutation types. The correlation between fluoroquinolone resistance and the individual mutation type was analyzed.

**Results:**

Resistance differences between ciprofloxacin and levofloxacin were found in 327 isolates of *K. pneumoniae* and *E. coli* in Harbin, China and in the isolates reported in PubMed publications. GyrA mutations were found in both susceptible and resistant isolates. For the isolates with QRDR mutations, the resistance rates to ciprofloxacin and levofloxacin were also statistically different. Among the 14 patterns of alterations, two single mutations (Ser83Tyr and Ser83Ile), and three double mutations (Ser83Leu+Asp87Asn, Ser83Leu+Asp87Tyr and Ser83Phe+Asp87Asn) were associated with both ciprofloxacin and levofloxacin resistance. Two single mutations (Ser83Phe and Ser83Leu) were related with ciprofloxacin resistance but not to levofloxacin. Resistance difference between ciprofloxacin and levofloxacin in isolates harboring mutation Ser83Leu+Asp87Asn were of statistical significance among all *Enterobacteriaceae* (*P*<0.001).

**Conclusions:**

Resistance rate to ciprofloxacin and levofloxacin were statistically different among clinical isolates of *Enterobacteriaceae* harboring GyrA mutations. Ser83Leu+Asp87Asn may account for the antimicrobial resistance difference between ciprofloxacin and levofloxacin.

## Background

Fluoroquinolones (FQs) have been used for the treatment of a vast variety of infections. They exert an antibacterial effect through inhibiting DNA synthesis by interacting with DNA gyrase and topoisomerase IV. Ciprofloxacin and levofloxacin are the two most frequently prescribed FQs. Ciprofloxacin was introduced into clinical use about three decades ago, and became widely used as a result of its effective activity against Gram-negative bacteria, especially the *Enterobacteriaceae*[1]. Compared with ciprofloxacin, levofloxacin has an increased activity against gram-positive bacteria.

FQ resistance mechanisms have mostly been related to specific mutations that lead to amino acid alterations in the quinolone resistance-determining regions (QRDRs) in GyrA, a subunit of DNA gyrase, by point mutations. The levofloxacin molecule has an additional C-8 methoxy substitution on the fluorinated quinoloic acid cores as compared to ciprofloxacin [2]. It has been shown that FQs containing the C-8 methoxy group, exhibit stronger antibacterial activity against bacteria that are resistant to quinolones due to GyrA mutation [3]. In vitro studies also showed that purified gyrase is more sensitive to FQs with C-8 substitutions [4,5]. Given the higher antibacterial activity of levofloxacin compared to that of ciprofloxacin, one would expect that clinical isolates would show a higher resistance rate to ciprofloxacin than to levofloxacin. Recent studies in various regions have consistently demonstrated that the percentage of clinical isolates resistant to ciprofloxacin is slightly higher than levofloxacin among *Enterobacteriaceae*[6-11]. Such a difference might partially due to the different breakpoints (4 μg/ml for ciprofloxacin and 8 μg/ml for levofloxacin) for the two drugs.

Our previous study has demonstrated that only part of the mutation patterns in quinolone resistance determining region (QRDR) of GyrA are related to ciprofloxacin resistance in clinical isolates of *K. pneumoniae*[12]. Other recent studies have also revealed that QRDR mutations at codon 83 and 87 in the *gyr*A gene are related to ciprofloxacin resistance, whereas those at amino acid codon 83 are related to levofloxacin resistance in *K. pneumoniae* isolates [13]. These results indicate that the mutation position within QRDR is linked with resistance to different classes of FQs. To clarify the effect of different amino acid mutation patterns of GyrA on the resistance difference between ciprofloxacin and levofloxacin, we investigated the drug resistance difference against two agents in clinical isolates of *K. pneumoniae* and *E. coli* in Harbin, China, and in the isolates in publications indexed by PubMed. We also studied the relationship between the patterns of QRDR mutations in *gyr*A among these isolates and the antimicrobial resistance difference against the two FQs.

## Methods

### Bacterial collection and antimicrobial susceptibility test

Between March 2009 and December 2009, one hundred and forty-four consecutive, non-repetitive isolates of *K. pneumoniae* and *E. coli* were collected from inpatients in four tertiary hospitals in Harbin, the provincial headquarter of Heilongjiang province, North China. The strains were isolated from inpatients in surgical wards, medical wards, intensive care units, and pediatric wards. Identification to the species level was confirmed with the API 20E system (bioMérieux, Marcy I’Etoile, France). All isolates were subjected to Randomly Amplified Polymorphism DNA (RAPD) analysis to eliminate any replicated strains. MICs to ciprofloxacin and levofloxacin were determined by microdilution method according to CLSI guidelines [14]. The MIC for each clinical isolate was measured at least in triplicate and the median of the all results was reported. Occasionally, MICs from independent experiments in the triplicate varied, but only by one step higher or lower in the dilution series. In such cases, the MIC was measured in additional experiments, and the median MIC was reported.

### Amplification and sequencing of the *gyr*A fragments

Seventy-nine and 65 clinical isolates of *K. pneumoniae* and *E. coli* were randomly selected to determine the DNA sequence of QRDR of *gyr*A. Oligonucleotide primers used for amplification of fragments encompassing the QRDR were, *gyr*A-F (5^′^-TGCGAGAGAAATTACACC) and *gyr*A-R (5^′^-AATATGTTCCATCAGCCC). The *gyr*A gene fragments were amplified from crude cell lysates. PCR products were purified and then sequenced in both directions by an automated DNA sequencer (ABI PRISM 373; Applied Biosystems, Foster City, CA) with the same primers used in the PCR amplification. The nucleotide sequences and the deduced amino acids were compared with that of *K. pneumoniae* ATCC13883 (GenBank DQ673325) and *E. coli* K-12 (GenBank NC_010473.1) using the online ClustalW2 multiple sequence alignment program.

### Identification of alteration types in GyrA required for ciprofloxacin resistance

MICs of ciprofloxacin and levofloxacin, and alteration types in Ser-83 and Asp-87 in GyrA of *K. pneumoniae* and *E. coli* were reviewed in the publications indexed in PubMed. All the amino acid substitutions associated with the susceptibility to ciprofloxacin and levofloxacin, including the changes found in this study, were summarized. All the strains involved in the publications and those in this study were resistant to levofloxacin and ciprofloxacin based on the CLSI criteria [14]. Amino acid substitution profiles were analyzed for association with FQ resistance.

### Nucleotides sequence accession numbers

The partial sequences of the variant *gyr*A genes in the clinical isolates have been submitted to GenBank under accession numbers EU430280 through EU430289, and JQ694717 through JQ694722.

### Statistical methods

Resistance rates among the isolates were analyzed by paired chi-square test. The correlation between fluoroquinolone resistance and the individual alteration were analyzed by Fisher’s exact test or Pearson chi-square test (IBM SPSS 13.0 statistical package). *P*<0.05 was considered as statistically significant.

## Results

### Drug resistance difference between levofloxacin and ciprofloaxin

A total of 144 isolates of *K. pneumoniae* and *E. coli* were collected in Harbin. According to CLSI breakpoints [14], resistance rates to ciprofloxacin and levofloxacin were 65.3% and 54.9%, respectively. Among 79 isolates of *K. pneumoniae*, 41 (51.9%) and 32 (40.5%) were resistant to ciprofloxacin and levofloxacin, respectively. Among the 65 isolates of *E. coli*, 53 (81.9%) and 47 (72.3%) isolates were resistant to ciprofloxacin and levofloxacin, respectively. These results showed that resistance rates were higher for ciprofloxacin than levofloxacin among the *K. pneumoniae* and *E. coli* isolated from Harbin.

A total of 183 isolates of *K. pneumoniae* and *E. coli* reported by publications indexed by PubMed were chosen in this study because they had both the MIC values of ciprofloxacin and levofloxacin, and DNA sequences for the QRDR of the *gyr*A gene. This included 34 strains of *K. pneumoniae* obtained from the hospitals under SENTRY antimicrobial surveillance program in Europe [6], in which 14 (41.2%) and 6 (17.6%) isolates were resistant to ciprofloxacin and levofloxacin, respectively. Among 149 isolates of *E. coli* reported in seven publications [10,15-20], the resistance rate to ciprofloxacin was 86.6% and that to levofloxacin were 86.6%. The overall resistance rates to ciprofloxacin and levofloxacin for these *Enterobacteriaceae* were 78.1% and 73.8%, respectively.

A total of 327 isolates were sampled from both our study (144) and the PubMed publications (183) and the accumulative resistance rates were 72.5% for ciprofloxacin and 65.7% for levofloxacin.

### QRDR mutations

Among all the 327 isolates of *Enterobacteriaceae*, QRDR mutations in the *gyr*A gene were identified showing 14 distinct patterns (Table 1). For *K. pneumoniae* isolates with QRDR mutation in *gyr*A gene, MICs for the two quinolones were either higher or lower than breakpoints specified by CLSI (Table 1). It showed that there were both resistant and susceptible isolates to the two FQs among isolates with QRDR mutation.

**Table 1:** ***Gyr*A mutation and the susceptibilities to levofloxacin and ciprofloxacin in 327 isolates of *Enterobacteriaceae***

**Mutation**	**No. of isolates**^**a**^	**CLSI levofloxacin MIC breakpoint**		**P value**	**OR**^**d **^**(CI**^**e **^**-95%)**	**CLSI ciprofloxacin MIC breakpoint**		**P value**	**OR (CI-95%)**
		**≥8 μg/ml**^**b**^	**<8 μg/ml**			**≥4 μg/ml**	**<4 μg/ml**^**c**^		
No mutation	66 (29)	4 (0)	43 (29)			5 (0)	61(29)		
Mutation	261(154)	210(136)	51(18)	<0.001	45.365(15.562-132.246)	232(143)	29(11)	<0.001	97.600 (36.262-262.690)
Ser83Tyr	10 (9)	2 (1)	8 (8)	<0.05	2.688(0.419-17.222)	6 (5)	4 (4)	<0.001	18.300(3.848-87.038)
Ser83Ile	10 (1)	8(1)	2 (0)	<0.001	43.000 (6.710-275.554)	9 (1)	1 (0)	<0.001	109.8 (11.478-1050.351)
Ser83Leu+Asp87Asn	169 (96)	152 (96)	17 (0)	<0.001	96.118 (30.722-300.715)	163 (96)	6 (0)	<0.001	331.433 (97.582-1125.700)
Ser83Leu+Asp87Tyr	38 (34)	37(33)	1 (1)	<0.001	397.750(42.558-3717.362)	37 (33)	1(1)	<0.001	451.400(50.745-4015.373)
Ser83Phe+Asp87Asn	4(4)	3(3)	1(1)	<0.001	32.250(2.689-386.757)	4(4)	0	<0.001	0
Ser83Phe	7 (6)	1(0)	6 (6)	>0.05	1.792(0.171-18.822)	3 (2)	4 (4)	<0.05	9.15(1.586-52.799)
Ser83Leu	14(1)	4(0)	10 (1)	>0.05	4.300(0.915-20.205)	5 (0)	9 (1)	<0.05	6.778(1.633-28.139)
Asp87Asn	1 (0)	0	1 (0)	-	-	1 (0)	0	-	-
Ser83Thr	3 (0)	0	3 (0)	-	-	0	3 (0)	-	-
Ser83Ala	1(1)	0	1(1)	-	-	0	1(1)	-	
Ser83Phe+Asp87Tyr	1(1)	0	1(1)	-	-	1(1)	0	-	-
Ser83Tyr+Asp87Tyr	1 (1)	1 (1)	0	-	-	1 (1)	0	-	-
Ser83Leu+Asp87His	1(0)	1(0)	0	-	-	1(0)	0	-	-
Ser83Phe+Asp87Ala	1(0)	1(0)	0	-	-	1(0)	0	-	-
Total	327(183)	214 (135)	113 (48)			237 (143)	90 (40)		

a: Numbers in brackets are isolates number in reference.

b: CLSI breakpoint for levofloxacin resistance.

c: CLSI breakpoint for ciprofloxacin resistant.

-: limited numbers of isolates, no statistical analysis could be done.

d: OR: odd ratio.

e: CI: Confidence Interval.

Statistical multivariate analysis showed two single mutations (Ser83Tyr and Ser83Ile) and three double mutations (Ser 83Leu+Asp87Asn, Ser83Leu+Asp87Tyr and Ser83Phe+Asp87Asn) to be related to both ciprofloxacin and levofloxacin resistance (all *P* value less than 0.05 or 0.001), compared with the isolates without mutations. Two single mutations (Ser83Phe and Ser83Leu) were related to ciprofloxacin resistance (*P*<0.05) but not to levofloxacin resistance (*P*>0.05). All the remaining 7 mutations were not related to resistance to any of the two FQs. The MICs to quinolones of isolates with the above mutations were also elevated but were not as high as those of isolates resistant to these drugs. The OR (odds ratio) value in Table 1 indicated the correlation of the two drugs with mutations. A higher OR value reflected a higher degree of correlation.

### Differences in antimicrobial resistance to ciprofloxacin and levofloxacin of isolates in relation to *gyr*A alterations

**Figure 1:**
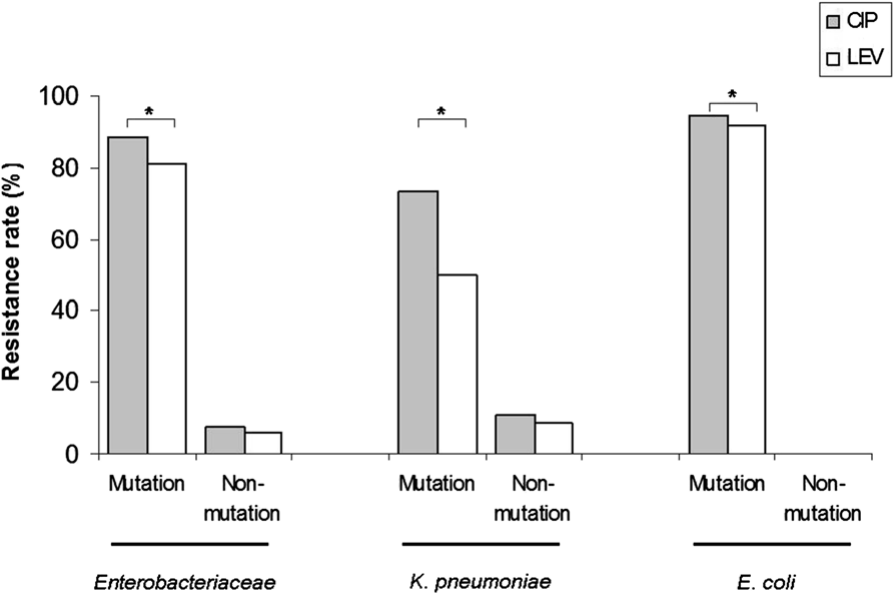
**Difference of resistant rate to ciprofloxacin and levofloxacin in *Enterobacteriaceae *with and without mutations in *gyr*A QRDR.**

As shown in Figure 1, by comparing the differences of drug-resistance to the two FQs in bacteria in relation to GyrA mutations, we found that the resistance difference between ciprofloxacin and levofloxacin in isolates with *gyr*A gene alterations were statistically significant among *E. coli*, *K. pneumoniae* or all *Enterobacteriaceae* (all *P*<0.05). However, no statistical significance were found among isolates without GyrA mutations (*P*>0.05).

We then investigated the resistance difference to ciprofloxacin and levofloxacin in isolates carrying different patterns of *gyr*A alterations. The results are shown in Table 2. Among 169 *Enterobacteriaceae* isolates with Ser83Leu+Asp87Asn mutation, the resistance difference to the two agents was of statistical significance (*P*<0.001). Comparatively, no statistical significance of the resistance to the two agents was found in isolates carrying any of the other types of mutations (*P*>0.05).

**Table 2:** **Resistance difference to ciprofloxacin and levofloxacin in *Enterobateriaceae *with various mutations in GyrA**

**Mutation**	**No. of isolates**^**a**^	**No. of isolates with different susceptibilities to ciprofloxacin and levofloxacin**^**b**^				**P value**
		**CIP-R LEV-R**	**CIP-R LEV-S**	**CIP-S LEV-R**	**CIP-S LEV-S**	
No mutation	66 (29)	4 (0)	1 (0)	0	61(29)	>0.05
mutation	261(154)	211(136)	21(7)	0	30(12)	9.54E-07
Ser83Tyr	10 (9)	2 (1)	4(4)	0	4 (4)	>0.05
Ser83Ile	10 (1)	8(1)	1(0)	0	1 (0)	>0.05
Ser83Phe	7 (6)	1(0)	2(2)	0	4 (4)	>0.05
Asp87Asn	1 (0)	0	1(0)	0	0	-
Ser83Thr	3 (0)	0	0	0	3 (0)	-
Ser83Ala	1(1)	0	0	0	1(1)	-
Ser83Leu	14(1)	4(0)	1(0)	0	9 (1)	>0.05
Ser83Leu+Asp87Asn	169 (96)	152 (96)	11(0)	0	6 (0)	1.00E-03
Ser83Leu+Asp87Tyr	38 (34)	37(33)	0	0	1 (1)	>0.05
Ser83Phe+Asp87Asn	4(4)	3(3)	1(0)	0	0	-
Ser83Phe+Asp87Tyr	1(1)	0	0	0	1(1)	-
Ser83Tyr+Asp87Tyr	1 (1)	1 (1)	0	0	0	-
Ser83Leu+Asp87His	1(0)	1(0)	0	0	0	-
Ser83Phe+Asp87Ala	1(0)	1(0)	0	0	0	-
Total	327(183)	214 (135)	22(7)	0	91 (41)	4.77E-07

a: Numbers in brackets are isolates number in references.

b: CIP, ciprofloxacin; LEV, levofloxacin.

Of 10 mutation patterns identified in *K. pneumoniae*, 9 were either related to ciprofloxacin and levofloxacin drug-resistance or simultaneously unrelated to ciprofloxacin and levofloxacin drug-resistance. However, *K. pneumoniae* isolates with Ser83Tyr substitution were related to ciprofloxacin drug-resistance but unrelated to levofloxacin (Table 1). Except for two mutations identified in one bacterial isolate, all the other mutations in *E. coli* were related to ciprofloxacin and levofloxacin simultaneously.

## Discussion

Previous studies have found that ciprofloxacin showed a higher number of resistant strains than levofloxacin among the clinical isolates of *Enterobacteriaceae*[5-7,9-11,17]. In the present investigation, we also observed similar data among the clinical isolates of *K. pneumoniae* and *E. coli*. Moreover, we found that resistance difference to ciprofloxacin and levofloxacin was of statistical significance among the isolates with QRDR mutations. Such differences might be mainly addressed in terms of the different CLSI resistant breakpoints (4 μg/ml for ciprofloxacin and 8 μg/ml for levofloxacin), which are supposed to integrate more strains with an elevated MIC for ciprofloxacin into the resistant bacterial population.

Nevertheless, a cluster of studies have shown that gyrase had different sensitivity and affinity to ciprofloxacin and levofloxacin, owing to the molecular structural difference of the two fluoroquinolones [1,21-23]. One can postulate that different amino acid substitution in QRDR might play different roles in resistance to the two agents. In the present work, we investigated the relationship between the drug-resistance between ciprofloxacin and levofloxacin and specific mutations of *gyr*A gene. In combination with relevant studies reported in PubMed, we analyzed the relationship between QRDR mutations and the resistance to ciprofloxacin and levofloxacin. Of the 14 patterns of QRDR mutations found in 327 *Enterobacteriaceae* isolates, 5 patterns of mutations were related to ciprofloxacin and levofloxacin resistance. Two single mutations (Ser83Phe and Ser83Leu) were related to ciprofloxacin resistance but not to levofloxacin resistance. None of the remaining 7 patterns of mutation were found to be related to resistance to any one of the two FQs. Further study about the interactions between various amino acid substitutions and different FQs molecules would give a comparison of amino acid preference for FQ, and that a certain amino acid substitution may bring about a varied resistance to different FQs.

In our study, among 5 QRDR mutations in the *gyr*A gene related to both ciprofloxacin and levofloxacin resistance, isolates with Ser83Leu+Asp87Asn mutation were shown to have statistically different drug resistance between ciprofloxacin and levofloxacin. It could be inferred that such resistance difference between the two drugs was mainly due to Ser83Leu+Asp87Asn mutation. As for the other types of effective mutations, no statistically significant difference was found in drug resistance difference between the two FQs among the isolates with these mutations.

Additionally, there were four resistant isolates without *gyr*A mutations in this study. None of them were found to harbor plasmid-mediated quinolone resistance genes. Further examination about the active efflux or permeability changes for the four strains is needed.

Taken together, we found that there is a statistical difference in antimicrobial resistance between ciprofloxacin and levofloxacin among clinical isolates of *Enterobacteriaceae*. Based upon the effectiveness of resistance, mutation patterns of QRDR can be divided into effective mutation and neutral mutation. Ser83Leu+Asp87Asn mutation, as an effective mutation, caused the drug resistance difference between ciprofloxacin and levofloxacin in *Enterobacteriaceae* isolates.

## Conclusions

Resistance rate to ciprofloxacin and levofloxacin were statistically different among clinical isolates of *Enterobacteriaceae* harboring *gyr*A mutations. Ser83Leu+Asp87Asn substitutions may account for the antimicrobial resistance difference between ciprofloxacin and levofloxacin.

## Competing interests

All authors declare that they have no competing interests.

## Authors’ contributions

YF and FZ participated in the design, conduction, analysis and interpretation of the study. YF and WZ were involved in all phases of the experiment. HW and SZ were involved in the manipulation of clinical isolates. FM and XC were involved in the DNA sequencing. YC and HX conducted the statistical analysis. YF wrote the manuscript. FZ reviewed the initial and final drafts of the manuscript. All authors read and approved the final manuscript.

## Pre-publication history

The pre-publication history for this paper can be accessed here:

http://www.biomedcentral.com/1471-2334/13/8/prepub
